# Combined association of chronic disease and low skeletal muscle mass with physical performance in older adults in the Sarcopenia and Translational Aging Research in Taiwan (START) study

**DOI:** 10.1186/s12877-015-0011-6

**Published:** 2015-02-18

**Authors:** Chia-Ing Li, Tsai-Chung Li, Wen-Yuan Lin, Chiu-Shong Liu, Chih-Cheng Hsu, Chao A Hsiung, Ching-Yu Chen, Kuo-Chin Huang, Chih-Hsing Wu, Ching-Yi Wang, Cheng-Chieh Lin, for the Sarcopenia and Translational Aging Research in Taiwan (START) Team

**Affiliations:** Department of Medical Research, China Medical University Hospital, Taichung, Taiwan; School of Medicine, China Medical University, 91 Hsueh-Shih Road, Taichung, 40421 Taiwan; Graduate Institute of Biostatistics, College of Public Health, China Medical University, Taichung, Taiwan; Department of Family Medicine, China Medical University Hospital, Taipei, Taiwan; Institute of Population Health Sciences, National Health Research Institutes, Miaoli County, Taiwan; Department of Health Services Administration, China Medical University, Taichung, Taiwan; Department of Family Medicine, National Taiwan University, Taipei, Taiwan; Department of Family Medicine, National Cheng Kung University Hospital, Tainan, Taiwan; School of Physical Therapy & Center for Education and Research on Geriatrics and Gerontology, Chung Shan Medical University, Taichung, Taiwan; Department of Healthcare Administration, College of Medical and Health Science, Asia University, Taichung, Taiwan

**Keywords:** Chronic disease, Low muscle mass, Physical performance

## Abstract

**Background:**

Multiple chronic conditions and low skeletal muscle mass are common features of aging that are detrimental to physical performance. This study evaluates the simultaneous impact of these conditions on physical performance in older adults.

**Methods:**

Five studies from 2003 to 2012 were pooled to include 2,398 adults aged ≥65 years with diagnosed chronic diseases measured by self-administered questionnaire. Low muscle mass was defined as an appendicular skeletal muscle mass index less than that of the sex-specific lowest quintile in the population of older adults. Poor physical performances were defined as the lowest quintile of grip strength and gait speed in the population of older adults and the slowest sex-specific 20% of Timed Up and Go (TUG) test at each study site. Chi-squared and logistic regression tests were applied for data analysis.

**Results:**

Mean age of the study participants, of whom approximately 50% were men, was 74.3 years. Slow gait speed was nearly three times more likely to occur in the presence of low muscle mass coupled with chronic disease than in the absence of both factors after adjustment for study site, age, sex, education, marital status, body mass index, tobacco and alcohol use, and comorbidities. The independent effect of low muscle mass was generally stronger than that of each disease. Participants with more than two chronic diseases and low muscle mass were significantly more likely to perform poorly than those with no risk factors (odds ratio [OR] = 2.51 in patients with low grip strength, OR = 3.89 in patients with low gait speed, and OR = 3.67 in patients with poor TUG test scores, all P < 0 .05) after adjustment.

**Conclusions:**

The combined association of chronic disease and low skeletal mass with physical performance was stronger than the effect of either factor alone.

## Background

The proportion of older adults (>65 y) in the overall population is growing in numerous countries [1]. With aging comes impaired physical performance, which is correlated with incident functional limitation in daily activities [2,3] and is a predictor of adverse health effects such as hospitalization, severe limitation of mobility, and death [4]. To prevent such impairment, individuals at high risk can be identified by determining the factors contributing to physical performance. Studies have indicated that low muscle mass and chronic disease are linked to poor physical performance [5-12] and that age-related loss of skeletal muscle mass is a common phenomenon among older adults [12]. One study reported the prevalence of low muscle mass to increase from 8.9% in women aged 76–80 years to 10.9% at 86–95 years of age [13]. Muscle mass has been reported to decline 1–2% annually after age 50 years [14], which contributes to a decline in muscle strength [5,15]. The link between muscle mass and physical performance remains controversial. Some studies have indicated that low muscle mass is associated with low grip strength and poor mobility in older men and women [5,6,16], whereas others have observed no such association [15,17,18].

Previous studies have reported that low muscle strength and impaired physical performance have been linked to chronic diseases, including diabetes [9,10,19], hypertension [8], arthritis [12], and osteoporosis [11]. Older people with diabetes have less muscle strength and a slower gait than those without diabetes [9,10,19], and those with arthritis have less muscle strength than the general population at a similar age [12]. One prospective study revealed that having two or more chronic diseases is associated with a greater decline in grip strength than having no chronic disease [8]. The co-occurrence of low muscle mass and chronic disease is common as people age, and both factors are associated with poor physical function. However, the combined association of chronic disease and low muscle mass with physical function has not yet been examined. Therefore, in this study we explored the combined association of these factors with physical function in older adults.

## Methods

### Participants

Our pooled dataset was derived from 5 cohort studies of community-dwelling older adults from 2003 to 2012 that constitute the study entitled Sarcopenia and Translational Aging Research in Taiwan (START) [20]. We used 4 studies, excluding one study in which body composition was not measured. The four individual cohort studies comprising the present study were Healthy Aging Longitudinal Study in Taiwan (HALST) (n = 990), Taichung Community Health Study for the Elderly (TCHS-E) (n = 1042), Tianliao Old People (TOP) study (n = 549), and Comprehensive Geriatric Assessment and Frailty Study of Elderly Outpatients (CGAFSEO) (n = 431) [21-23]. These studies recruited subjects representative of older residents living in the northern (HALST, CGAFSEO), central (TCHS-E), and southern (HALST, TOP) regions of Taiwan. For the studies that are still ongoing (follow-up phase), only baseline data are reported here. All of the cohort studies had written informed consent, and were approved by the respective institutional review boards (HALST and CGAFSEO by Medical Research Ethics Committee of National Health Research Institutes and Research Ethics Committee of the National Taiwan University Hospital, TCHS-E by Institutional Review Board of China Medical University, TOP by Research Ethics Committee of National Cheng Kung University, and IPFCEH by Research Ethics Committee of Hualien Tzu Chi Hospital).

**Figure 1:**
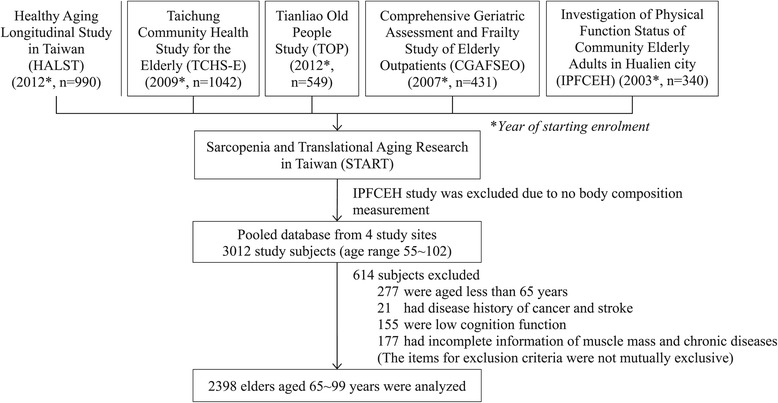
**Flowchart of study subjects.**

Our pooled data were therefore for 3,012 subjects ranging in age from 55 to 102 years (Figure 1). We excluded subjects aged younger than 65 years; those with cancer, stroke, and low cognitive function; and those with incomplete information on muscle mass and chronic disease. The exclusion criteria for stroke and cancer were applied on the basis of patients’ self-reported illness diagnosed by physicians. Subjects’ cognitive status was measured with the Mini-Mental State Examination (MMSE) at three study sites and the Short Portable Mental Status Questionnaire (SPMSQ) at one study site. For studies using the MMSE, low cognition was defined as a score of <18 in subjects with ≤9 years of education or a score of <25 in subjects with >9 years of education. For the study using the SPMSQ, low cognition was defined as four or more incorrect answers in subjects with ≤9 years of education, three or more incorrect answers in subjects with high school education, and two or more incorrect answers in subjects with senior high school education or higher. Ultimately, 2,398 community-dwelling older adults with a mean age of 74.3 ± 6.1 years were analyzed in this study. The study was approved by the institutional review boards at each site, and written informed consent was provided by each participant.

### Measurements

#### Low muscle mass

All participants underwent a standardized procedure for measuring body composition with an eight-contact-electrode bioelectrical impedance analysis device (Tanita BC-418; Tanita Corp., Tokyo, Japan) [24]. The device measures whole-body and segmental impedance (±1Ω) at a frequency of 50 kHz and provides valid estimates of muscle mass (kg) in all four extremities [25]. Appendicular muscle mass was calculated as the sum of the estimated muscle mass of the arms and legs. Appendicular muscle mass index (ASMI) normalized to height was defined as the ratio of appendicular muscle mass (kg) to height squared (m^2^) [18,25]. Muscle mass was considered low if ASMI was in the lowest 20% of the sex-specific distribution in the study population (7.11 kg/m^2^ for men and 5.63 kg/m^2^ for women).

#### Physical performance

Functional performance was assessed using the hand grip strength test, gait speed, and Timed Up and Go (TUG) test. Hand grip strength (kg) was measured using a standard calibrated hand dynamometer. The average grip strength of dominant hand was used in analysis. Participants who ranked in the lowest 20% for sex and body mass index (BMI)-specific distribution were considered to have low grip strength [26]. To measure gait speed, the participants walked at their typical pace over a fixed distance ranging from 3 to 5 m at different study sites [27]. Gait speed was determined by dividing the walking distance by total walking time, and participants ranking in the lowest 20% for sex- and height-specific distributions were considered to have a slow gait [26]. The cutoff points of gait speed and hand grip strength were published in our previous study [20]. Three study sites performed the TUG test, in which each participant stood in front of a chair of standard height, walked 3 m, turned, and returned to a sitting position in the chair [28]. At two of the three sites, subjects were asked to complete the TUG test at their usual pace, and at the third, subjects were asked to perform the test as quickly as possible [29]. Because the distributions of time required to complete the TUG test differed at different sites, site-specific distributions for TUG test results were used. Participants for whom the time required to complete the TUG test ranked in the highest 20% for sex by site were considered to have poor functional mobility.

#### Chronic disease

Data on chronic disease were collected using a self-report questionnaire. Patients were asked whether they had diabetes, hypertension, heart disease, chronic obstructive pulmonary disease (COPD), chronic kidney disease (CKD), arthritis, or osteoporosis. Comorbidity was measured as the total number of 7 chronic diseases and categorized as 0, 1, and 2 or more.

#### Covariates

Data on the sociodemographic characteristics and health-related behaviors of the participants, including age, sex, education, marital status, obesity status, smoking status, alcohol use, and physical activity level, were collected using a questionnaire. BMI, calculated as body weight (kg) divided by the square of height (m), was used to classify obesity status as underweight (<18.5 kg/m^2^), normal (18.5–24 kg/m^2^), overweight (24–26.9 kg/m^2^), or obese (≥27 kg/m^2^), as defined by the Taiwan Department of Health [30]. Energy expenditure through physical activity was derived from a standardized questionnaire. Information on various leisure activities and time spent per week performing each activity were obtained. Physical-activity energy expenditure per person was calculated as the total amount of time spent in each activity multiplied by typical energy expenditure and expressed in kilocalories expended per kilogram of body weight per week (kcal/kg/week) [31]. Participants were divided into site- and sex-specific tertiles of total energy expenditure from physical activity.

### Statistical analysis

To examine the correlations between physical performance and covariates (sociodemographic characteristics, obesity status, and health behaviors) and chronic disease, we compared the proportion in each covariate and chronic disease between participants with and without poor physical performance using the chi-square test. To test the combined association of chronic disease and muscle mass on physical performance, five common chronic diseases, each with a prevalence of >10% in older adults, were analyzed: diabetes, hypertension, heart disease, arthritis, and osteoporosis (COPD and CKD were excluded). Unadjusted odds ratio (OR) and 95% confidence interval (CI) were used to assess the combined association of chronic disease and muscle mass with physical performance, and multiple logistic regression was used to adjust for covariates. Two-sided *P* values were calculated and statistical significance set at *P* < 0.05. Analyses were performed using SAS® software version 9.3 (SAS Institute Inc., Cary, NC, USA).

## Results

Table 1 presents sociodemographic factors, health behaviors, chronic diseases, and low muscle mass categorized by low grip strength, gait speed, and TUG test performance. The combined association of chronic disease and low muscle mass with physical performance is presented in Table 1. The independent effect of low muscle mass significantly increased the odds of low grip strength among participants without hypertension (OR = 1.93), arthritis (OR = 1.72), or osteoporosis (OR = 1.65). We did not observe an independent effect of each disease on grip strength. While diabetes and low muscle mass coexisted, the increased odds of low grip strength (OR = 2.43) was observed in participants.

**Table 1:** **Effects of socio-demographic factors, health behavior, chronic disease, and muscle mass on physical performance**

	**Grip strength**			**Gait speed**			**TUG test performance**		
	**Normal**	**Low**		**Normal**	**Low**		**Normal**	**Low**	
**Variables**	**n (%)**	**n (%)**	**p-value**	**n (%)**	**n (%)**	**p-value**	**n (%)**	**n (%)**	**p-value**
**Socio-demographic variables**									
Age (years)			*<0.001*			*<0.001*			*<0.001*
65-69	602 (30.54)	61 (14.91)		611 (31.58)	51 (11.78)		553 (35.96)	34 (8.92)	
70-74	609 (30.90)	80 (19.56)		599 (30.96)	89 (20.55)		467 (30.36)	81 (21.26)	
75-79	435 (22.07)	100 (24.45)		427 (22.07)	106 (24.48)		301 (19.57)	100 (26.25)	
≥80	325 (16.49)	168 (41.08)		298 (15.40)	187 (43.19)		217 (14.11)	166 (43.57)	
Gender			*<0.001*			0.579			0.959
Woman	964 (48.91)	236 (57.70)		968 (50.03)	223 (51.50)		793 (51.56)	197 (51.71)	
Man	1007 (51.09)	173 (42.30)		967 (49.97)	210 (48.50)		745 (48.44)	184 (48.29)	
Education years			*0.003*			*<0.001*			*<0.001*
≤6	998 (51.23)	241 (59.21)		969 (50.55)	263 (61.74)		619 (40.62)	213 (57.10)	
>6	950 (48.77)	166 (40.79)		948 (49.45)	163 (38.26)		905 (59.38)	160 (42.90)	
Marital status			0.927			*0.002*			0.7480
Married	1718 (96.35)	282 (96.25)		1634 (96.97)	359 (93.73)		1250 (95.64)	299 (95.22)	
Unmarried	65 (3.65)	11 (3.75)		51 (3.03)	24 (6.27)		57 (4.36)	15 (4.78)	
BMI (kg/m^2^)			0.737			*<0.001*			*<0.001*
<18.5	66 (3.35)	15 (3.67)		57 (2.95)	24 (5.54)		42 (2.73)	17 (4.46)	
18.5-24	828 (42.01)	162 (39.61)		832 (43.00)	155 (35.80)		665 (43.24)	141 (37.01)	
24-27	633 (32.12)	131 (32.03)		630 (32.56)	126 (29.10)		506 (32.90)	105 (27.56)	
≥27	444 (22.53)	101 (24.69)		416 (21.50)	128 (29.56)		325 (21.13)	118 (30.97)	
**Health behavior**									
Smoking			0.150			0.641			0.723
No	1820 (92.34)	386 (94.38)		1793 (92.66)	404 (93.30)		1429 (92.91)	352 (92.39)	
Yes	151 (7.66)	23 (5.62)		142 (7.34)	29 (6.70)		109 (7.09)	29 (7.61)	
Alcohol drink			*0.001*			*0.010*			*0.016*
No	1610 (81.68)	361 (88.26)		1583 (81.81)	377 (87.07)		1224 (79.58)	324 (85.04)	
Yes	361 (18.32)	48 (11.74)		352 (18.19)	56 (12.93)		314 (20.42)	57 (14.96)	
Physical activity ^a^			*<0.001*			*<0.001*			*<0.001*
Low	560 (29.61)	190 (48.10)		541 (29.01)	195 (47.68)		404 (27.54)	186 (51.24)	
Normal	627 (33.16)	127 (32.15)		621 (33.30)	135 (33.01)		504 (34.36)	106 (29.20)	
High	704 (37.23)	78 (19.75)		703 (37.69)	79 (19.32)		559 (38.10)	71 (19.56)	
**Chronic disease**									
Diabetes			*<0.001*			*<0.001*			*<0.001*
No	1590 (80.67)	287 (70.17)		1563 (80.78)	310 (71.59)		1232 (80.10)	266 (69.82)	
Yes	381 (19.33)	122 (29.83)		372 (19.22)	123 (28.41)		306 (19.90)	115 (30.18)	
Hypertension			*<0.001*			*<0.001*			*<0.001*
No	910 (46.17)	125 (30.56)		886 (45.79)	147 (33.95)		693 (45.06)	110 (28.87)	
Yes	1061 (53.83)	284 (69.44)		1049 (54.21)	286 (66.05)		845 (54.94)	271 (71.13)	
Heart disease			0.260			*<0.001*			*<0.001*
No	1604 (81.38)	323 (78.97)		1599 (82.64)	318 (73.44)		1246 (81.01)	275 (72.18)	
Yes	367 (18.62)	86 (21.03)		336 (17.36)	115 (26.56)		292 (18.99)	106 (27.82)	
COPD			0.225			0.706			0.294
No	1916 (97.21)	393 (96.09)		1879 (97.11)	419 (96.77)		1490 (96.88)	365 (95.80)	
Yes	55 (2.79)	16 (3.91)		56 (2.89)	14 (3.23)		48 (3.12)	16 (4.20)	
CKD			*0.010*			0.519			0.729
No	1836 (93.15)	366 (89.49)		1788 (92.40)	404 (93.30)		1421 (92.39)	350 (91.86)	
Yes	135 (6.85)	43 (10.51)		147 (7.60)	29 (6.70)		117 (7.61)	31 (8.14)	
Arthritis			*<0.001*			*<0.001*			*<0.001*
No	1528 (77.52)	259 (63.33)		1484 (76.69)	295 (68.13)		1166 (75.81)	251 (65.88)	
Yes	443 (22.48)	150 (36.67)		451 (23.31)	138 (31.87)		372 (24.19)	130 (34.12)	
Osteoporosis			0.212			*<0.001*			*0.001*
No	1618 (82.09)	325 (79.46)		1611 (83.26)	321 (74.13)		1264 (82.18)	283 (74.28)	
Yes	353 (17.91)	84 (20.54)		324 (16.74)	112 (25.87)		274 (17.82)	98 (25.72)	
**Muscle mass**									
Low ASMI			*0.009*			*<0.001*			*0.005*
No	1601 (81.23)	309 (75.55)		1588 (82.07)	311 (71.82)		1207 (78.48)	273 (71.65)	
Yes	370 (18.77)	100 (24.45)		347 (17.93)	122 (28.18)		331 (21.52)	108 (28.35)	

*Abbreviations:* TUG, Timed Up and Go; BMI, body mass index; COPD, chronic obstructive pulmonary disease; CKD: chronic kidney disease; ASMI: Appendicular Skeletal Muscle Mass Index.

^a^Physical activity categorized according to sex-specific tertiles of physical activity.

The combined association of low muscle mass and chronic disease with gait speed exceeded the association of each factor alone. Independent associations of low muscle mass and slow gait were observed when diabetes (OR = 2.03), heart disease (OR = 1.73), hypertension (OR = 2.20), arthritis (OR = 1.88), or osteoporosis (OR = 1.90) was considered. An independent effect of chronic disease on gait speed was observed, with higher odds of slow gait significantly associated with diabetes, hypertension, and arthritis (OR = 1.76, 1.39, and 1.47, respectively; *P* < 0.05 for all). The likelihood of slow gait was far higher in the presence of both low muscle mass and chronic disease (OR = 2.73 for diabetes, OR = 2.97 for heart disease, OR = 2.38 for hypertension, OR = 3.06 for arthritis, and OR = 2.78 for osteoporosis, (*P* < 0.05) than in the absence of both factors (Table 2).

**Table 2:** **Combined association of chronic disease and low muscle mass with physical performance**

**Chronic disease**	**Low ASMI**	**Total n**	**Low grip strength**			**Low gait speed**			**Low TUG test performance**		
			**n (%)**	**OR**_**crud**_ **(95%CI)**	**OR**_**adj**_ **(95%CI)**	**n (%)**	**OR**_**crud**_ **(95%CI)**	**OR**_**adj**_ **(95%CI)**	**n (%)**	**OR**_**crud**_ **(95%CI)**	**OR**_**adj**_ **(95%CI)**
Diabetes											
No	No	1497	211 (14.09)	1.00	1.00	212 (14.16)	1.00	1.00	179 (11.96)	1.00	1.00
No	Yes	396	76 (19.19)	*1.46 (1.09, 1.95)*	1.52 (0.93, 2.48)	98 (24.75)	*1.99 (1.52, 2.61)*	*2.03 (1.31, 3.12)*	87 (21.97)	*1.63 (1.22, 2.18)*	*1.65 (1.02, 2.68)*
Yes	No	426	98 (23)	*1.82 (1.39, 2.38)*	1.36 (0.95, 1.96)	99 (23.24)	*1.85 (1.42, 2.42)*	*1.76 (1.25, 2.47)*	94 (22.07)	*1.93 (1.45, 2.57)*	*1.90 (1.29, 2.79)*
Yes	Yes	79	24 (30.38)	*2.64 (1.60, 4.35)*	*2.43 (1.14, 5.17)*	24 (30.38)	*2.76 (1.67, 4.57)*	*2.73 (1.33, 5.59)*	21 (26.58)	*2.32 (1.36,3.97)*	1.95 (0.87, 4.37)
Heart disease											
No	No	1581	246 (15.56)	1.00	1.00	237 (14.99)	1.00	1.00	202 (12.78)	1.00	1.00
No	Yes	362	77 (21.27)	*1.47 (1.11, 1.96)*	1.56 (0.95, 2.56)	81 (22.38)	*1.65 (1.24, 2.19)*	*1.73 (1.09, 2.73)*	73 (20.17)	*1.38 (1.03, 1.87)*	1.38 (0.82, 2.35)
Yes	No	342	63 (18.42)	1.22 (0.90, 1.65)	1.19 (0.78, 1.83)	74 (21.64)	*1.57 (1.17, 2.10)*	1.23 (0.83, 1.80)	71 (20.76)	*1.59 (1.17, 2.16)*	1.43 (0.93, 2.21)
Yes	Yes	113	23 (20.35)	1.39 (0.86, 2.24)	1.94 (0.94, 3.99)	41 (36.28)	*3.18 (2.12, 4.78)*	*2.97 (1.65, 5.34)*	35 (30.97)	*2.35 (1.53, 3.61)*	*2.48 (1.32, 4.65)*
Hypertension											
No	No	797	83 (10.41)	1.00	1.00	92 (11.54)	1.00	1.00	62 (7.78)	1.00	1.00
No	Yes	248	42 (16.94)	*1.77 (1.19, 2.65)*	*1.93 (1.06, 3.52)*	55 (22.18)	*2.19 (1.51, 3.17)*	*2.20 (1.30, 3.72)*	48 (19.35)	*2.24 (1.48, 3.39)*	*2.31 (1.25, 4.25)*
Yes	No	1126	226 (20.07)	*2.16 (1.65, 2.83)*	1.27 (0.90, 1.79)	219 (19.45)	*1.86 (1.43, 2.42)*	*1.39 (1.00, 1.91)*	211 (18.74)	*2.53 (1.87, 3.44)*	*2.02 (1.37, 2.98)*
Yes	Yes	227	58 (25.55)	*2.95 (2.02, 4.29)*	1.72 (0.97, 3.05)	67 (29.52)	*3.23 (2.25, 4.62)*	*2.38 (1.43, 3.99)*	60 (26.43)	*3.26 (2.19, 4.85)*	*2.26 (1.27, 4.03)*
Arthritis											
No	No	1426	189 (13.25)	1.00	1.00	205 (14.38)	1.00	1.00	177 (12.41)	1.00	1.00
No	Yes	373	70 (18.77)	*1.52 (1.12, 2.05)*	*1.72 (1.05, 2.81)*	90 (24.13)	*1.89 (1.43, 2.50)*	*1.88 (1.22, 2.90)*	74 (19.84)	*1.38 (1.02, 1.87)*	1.28 (0.78, 2.10)
Yes	No	497	120 (24.14)	*2.09 (1.62, 2.70)*	1.30 (0.91, 1.86)	106 (21.33)	*1.62 (1.25, 2.11)*	*1.47 (1.05, 2.06)*	96 (19.32)	*1.56 (1.18, 2.06)*	1.18 (0.80, 1.75)
Yes	Yes	102	30 (29.41)	*2.79 (1.77, 4.39)*	1.60 (0.75, 3.40)	32 (31.37)	*2.81 (1.80, 4.38)*	*3.06 (1.52, 6.15)*	34 (33.33)	*2.87 (1.83, 4.50)*	*2.93 (1.41, 6.10)*
Osteoporosis											
No	No	1574	245 (15.57)	1.00	1.00	227 (14.42)	1.00	1.00	203 (12.9)	1.00	1.00
No	Yes	382	80 (20.94)	*1.44 (1.09, 1.91)*	*1.65 (1.02, 2.68)*	94 (24.61)	*1.94 (1.48, 2.55)*	*1.90 (1.23, 2.93)*	80 (20.94)	*1.44 (1.07, 1.92)*	1.44 (0.88, 2.35)
Yes	No	349	64 (18.34)	1.22 (0.90, 1.66)	1.03 (0.70, 1.51)	84 (24.07)	*1.89 (1.42, 2.51)*	1.39 (0.97, 1.99)	70 (20.06)	*1.59 (1.17, 2.17)*	1.31 (0.86, 1.98)
Yes	Yes	93	20 (21.51)	1.52 (0.91, 2.54)	1.35 (0.62, 2.98)	28 (30.11)	*2.56 (1.61, 4.08)*	*2.78 (1.41, 5.48)*	28 (30.11)	*2.32 (1.44, 3.73)*	*2.25 (1.07, 4.71)*

ASMI = Appendicular Skeletal Muscle Mass Index; BMI = body mass index; CI = confidence interval; OR = odds ratio; ORadj = OR adjusted for age, sex, education, BMI, site, tobacco, alcohol, marital status, physical activity, and other chronic diseases.

The combined association of chronic disease and low muscle mass with TUG score was stronger than the independent effect of either factor alone after adjustment for confounding factors. The ORs of a low TUG test score for low muscle mass alone were 1.65 when considering diabetes and 2.31 when considering hypertension (*P* < 0.05 for both), and the relative odds for diabetes and hypertension alone were 1.90 and 2.02, respectively (*P* < 0.05). The ORs of a low TUG score were significant in the presence of low muscle mass and heart disease (OR = 2.48), hypertension (OR = 2.26), arthritis (OR = 2.93), or osteoporosis (OR = 2.25) (*P* < 0.05 for all), but not in the presence of low muscle mass with diabetes (Table 2).

**Figure 2:**
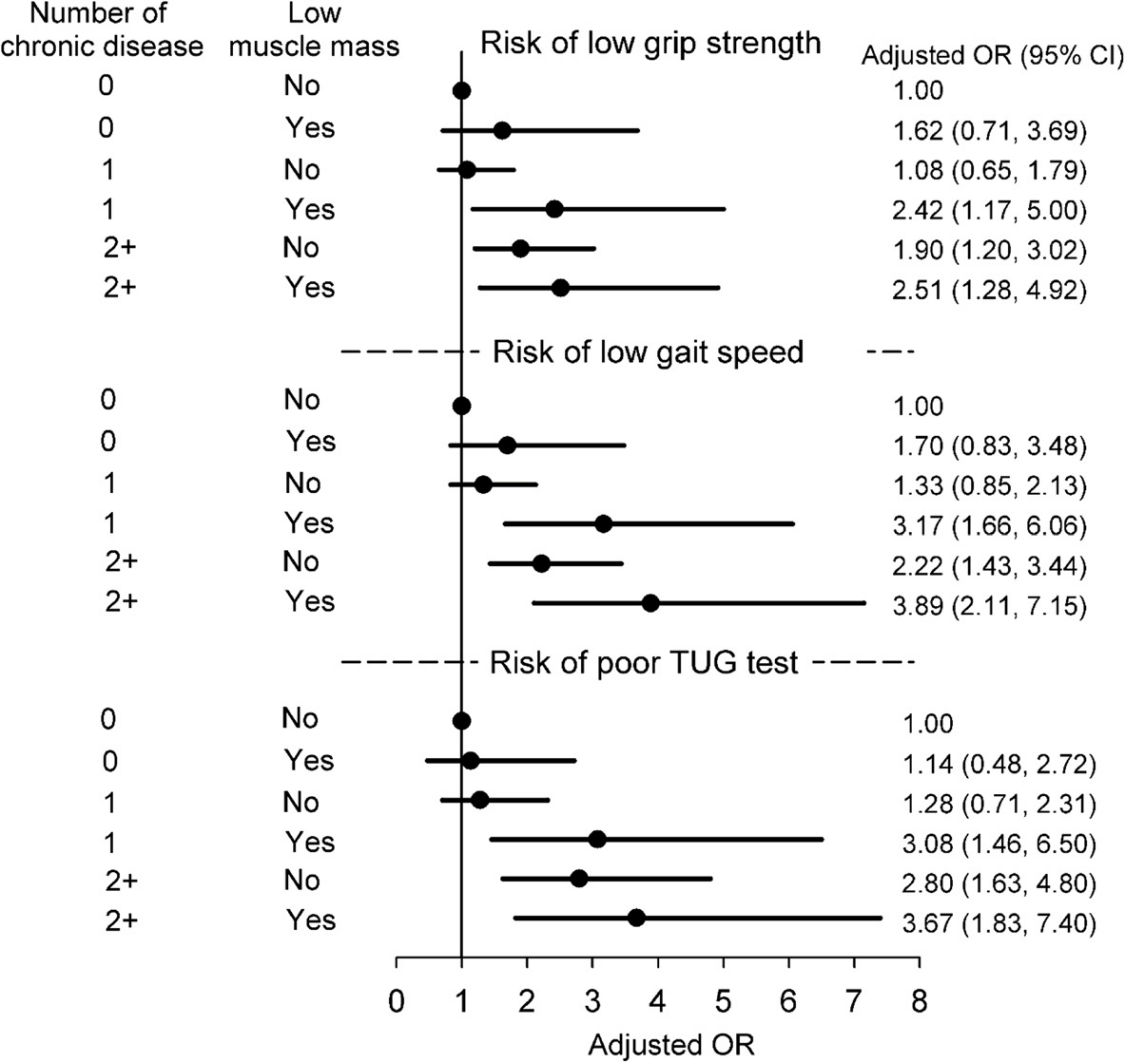
**Risk of poor physical performance according to combined association of number of chronic diseases and low muscle mass, adjusted for age, sex, education, BMI, study site, tobacco, alcohol, marital status, and physical activity.** BMI = body mass index.

Finally, we explored the combined association of multiple chronic diseases and low muscle mass. Participants with more than two chronic diseases and low muscle mass were more likely to perform poorly than those with no risk factors (OR = 2.51 for low grip strength, OR = 3.89 for slow gait, and OR = 3.67 for low TUG score, *P* < 0.05) after adjustment for confounding factors. Compared with participants with no risk factors, the odds of poor physical performance among those having one chronic disease and low muscle mass were higher than among participants with two or more chronic diseases and normal muscle mass (OR = 2.42 vs. 1.90 for low grip strength, OR = 3.17 vs. 2.22 for slow gait, and OR = 3.08 vs. 2.80 for a low TUG score; *P* < 0.05) (Figure 2).

## Discussion

In the present study, we examined the combined association of low muscle mass and chronic disease with physical performance in older adults. Participants with low muscle mass and chronic comorbidities had lower grip strength, slower gait, and lower TUG score than those with low muscle mass alone or chronic disease alone, even after we controlled for potential confounding factors. Furthermore, low muscle mass alone is more strongly associated with greater impairment in physical performance than is chronic disease alone.

Low muscle mass has been reported to be significantly associated with impaired physical performance in older adults [5,6,11,19,32-34]. A community-based study of 4,000 older adults linked low muscle mass to weaker grip strength in both sexes after adjusting for age [34]. Some studies have reported that older women with lower muscle mass exhibit slower gait [5,6,34]. In the United States, the third National Health and Nutrition Examination Survey reported that even after adjustment for confounding variables, low muscle mass hindered tandem-standing ability in older men [32]. A cross-sectional study analyzing 183 community-dwelling older adults using baseline data from a randomized control trial [11] and a community-based study of 409 older women [35] indicated that higher muscle mass is associated with a better TUG test score. These results provided evidence that muscle mass is highly correlated with physical performance in older adults.

Unlike the aforementioned studies, which reported the contribution of low muscle mass to physical performance, our study also assessed the effect of chronic disease while considering the combined contribution of muscle mass and chronic disease to the risk of poor physical performance in older adults. After we considered these diseases, our results were consistent with those of previous studies of the effect of muscle mass on gait speed [5,6,34]. Our data regarding the effect of muscle mass on grip strength were consistent with those of a previous study [34] when only hypertension, arthritis, and osteoporosis were considered, but were not significant for diabetes and heart disease. The effect on TUG score was similar when we considered heart disease, hypertension, arthritis, and osteoporosis, and was nonsignificant when we considered diabetes. The effects might not have been observed when chronic conditions were considered because of the presence of comorbidities in older adults.

We observed that older adults with diabetes or hypertension exhibited poor lower-extremity physical performance. This is consistent with the findings of two studies reporting that older diabetic adults had a slower gait and less muscle strength than older adults without diabetes [9,19], and with the results of a study that followed older adults for 18 years and found that higher systolic blood pressure was associated with a higher rate of decline in gait speed [36]. Poor muscle function in diabetic patients could be the result of neuropathic processes due to diabetic polyneuropathy, which involves motor neurons [37], and the catabolic effect of inflammation on muscles [38]. Our study further explored the combined association of low muscle mass and diabetes and our results showed that elders with low muscle mass and muscle dysfunction due to diabetic polyneuropathy and muscle inflammation may further worsen old adults’ physical activity performance including poor hand grip strength and low gait speed. The magnitude of strength of association for both low muscle mass and diabetes was much greater than that either for low muscle mass alone or diabetes alone. The hypertension effect may be attributable to vascular-related damage in the musculoskeletal and peripheral nervous systems [39]. Our study finding demonstrated that an elder with low muscle mass further suffering from vascular-related damage to the musculoskeletal and peripheral nervous system by hypertension may impede elders’ function performance including low gait speed and poor TUG test performance. Again, the strength of association for both low muscle mass and hypertension was stronger than that either for low muscle mass alone or hypertension alone. Our study was the first one to estimate the strength of joint association between low lean muscle mass and chronic diseases. These findings are of relevant to clinical management of chronic diseases in elders.

We also observed that older adults with arthritis exhibited slower gait and required a longer time to complete the TUG test. A meta-analysis of 185 studies with 101,049 participants reported that individuals with rheumatoid arthritis exhibited substantially less grip strength than similar-aged individuals in the general population [12]. A possible reason for the discrepancy between these results and those of the present study is that we considered arthritis to affect mainly the hips and knees, rather than the hands, because of the low the prevalence of symptomatic hand osteoarthritis in older Chinese adults (3.0% in men, 5.8% in women) [40]. The meta-analysis addressed grip strength but did not focus on the lower extremities.

Our study examined the independent as well as the combined associations of low muscle mass and various chronic diseases with physical performance. The independent association of low muscle mass was stronger than that of each disease with grip strength and gait speed, indicating that maintenance of muscle mass is crucial to improving physical performance. Participants with two or more chronic diseases and low muscle mass performed more poorly than those with no risk factors after we adjusted for confounding factors, which illustrates the importance of physical training for older adults with multiple chronic conditions. These findings suggest that clinicians, public health workers, and investigators can create strategies to prevent or slow the decline of physical performance in these groups.

One limitation of this study was the differences in measurement of physical performance at different study sites. Inter-observer consistency of measurement could not be verified. To control for the effect of this confounding factor, we used site-specific cutoff points to define performance status as normal or abnormal. The study site was added to multivariate models to control for the effect of this confounder on the relationship between muscle mass and comorbidities and physical performance. Another limitation was assessment of chronic disease by participant self-report without clinical confirmation of a diagnosis. This may have led to underestimation of disease prevalence; however, a previous study observed that self-reported data on common chronic conditions exhibit a moderate to strong agreement with medical records [41]. We may have underestimated the independent effect of chronic disease, or the combined association of chronic disease and low muscle mass, with physical performance through nondifferential misclassification.

## Conclusion

In older adults, the co-occurrence of low muscle mass and chronic disease contributed to a higher risk of impairment of physical performance than did either factor alone. The independent association between low muscle mass and physical-performance impairment was much stronger than that between each disease and physical-performance impairment.
